# Response: Commentary: The Nomenclature of Human White Matter Association Pathways: Proposal for a Systematic Taxonomic Anatomical Classification

**DOI:** 10.3389/fnana.2019.00091

**Published:** 2019-10-17

**Authors:** Emmanuel Mandonnet, Silvio Sarubbo, Laurent Petit

**Affiliations:** ^1^Department of Neurosurgery, Lariboisière Hospital, Paris, France; ^2^Division of Neurosurgery, Structural and Functional Connectivity Lab, Azienda Provinciale per i Servizi Sanitari, Trento, Italy; ^3^Groupe d'Imagerie Neurofonctionnelle, Institut des Maladies Neurodégénératives–UMR 5293, CNRS, CEA University of Bordeaux, Bordeaux, France

**Keywords:** white matter anatomy, association pathways, taxonomic classification, nomenclature, human brain, dissection, tractography

We thank Panesar and Fernandez-Miranda (2019) for their interest in our work (Mandonnet et al., 2018). However, based on their comments, there seems to be some misunderstanding. We should have more elaborated on the principles that grounded our classification. We aim at rationalizing the current terminology that everyone shares but which is anything but clear. To this end, we proposed a first principle, namely the taxonomic scheme. This way of organizing knowledge has been demonstrated to be very powerful in many areas of biological sciences. It allows to define at the same time broad categories (which we called “systems”), together with subcategories (which we called “tracts”). It allows the classification to be continuously revised as our knowledge of the exact trajectories of the connections becomes more and more accurate. We agree that numbering the different entities of a system by roman letters is not very informative (although please note that the numbering follows the anatomical orientation, from medial to lateral). However, this is the only way to make it easy to implement new knowledge. On the contrary, the anatomically-oriented attributes “dorsal” and “ventral” that are proposed by the authors are too restrictive consider for example the four subcomponents of the arcuate fasciculus described by the authors of the commentary (Fernández-Miranda et al., 2014).

With this general principle in mind, it seems clear that the arcuate fasciculus belong to the broad category of “superior longitudinal *system*,” as the central part of its trajectory (called the “stem,” in which all fibers converge before diverging again toward their destination) is in close relationship with the stems of the three superior longitudinal fasciculus. And we fully agree that the terminology “superior longitudinal *fasciculus*” should not be used for the arcuate fasciculus, as the whole shape of the tract is not longitudinal.

Regarding the comment about the cingulum, we do not really understand the criticism, as we fully concur with their statement. We were among the first to deny the existence of the SLF I as initially described by Makris (Makris et al., 2005; Maldonado et al., 2012), and we also agree, as explained in the paper, to assign to the mesial longitudinal system the fronto-parietal fibers located mesially to the corona radiata. However, in our view, the most dorsal and medial fibers of the SLF II could potentially connect the superior frontal lobe with the superior parietal lobule. Hence, we designated such a putative tract the SLF I.

Last but not least, we do not dispute the fact that the vertical (or aslant) temporo-parietal fasciculus is separated from the vertical occipital fasciculus. But again, the location and orientation of their stems makes intuitive their assignment to a common category that we called “posterior transverse system,” thus mirroring the anterior transverse system.

As imperfect and incomplete as it is, our taxonomic nomenclature has the merit to be a recursive, anatomically-based, and flexible approach to the hard task of classifying and designing the complex sets of connections of the human brain and we hope it could help naïve people to the field to assimilate more easily our current state of knowledge.

## Author Contributions

EM wrote the response to the commentary. SS and LP approved it for publication.

### Conflict of Interest

The authors declare that the research was conducted in the absence of any commercial or financial relationships that could be construed as a potential conflict of interest.
